# Association Between Osseous Shoulder Morphology and Pathoanatomical Characteristics of Calcific Deposits in Rotator Cuff Calcific Tendinitis

**DOI:** 10.3390/diagnostics15222908

**Published:** 2025-11-17

**Authors:** Andro Matković, Mia Grgić, Ines Trkulja, Marija Ivković, Thomas Ferenc, Nikolina Jurjević, Božidar Šebečić, Vinko Vidjak

**Affiliations:** 1 Department of Diagnostic and Interventional Radiology, Merkur University Hospital, 10000 Zagreb, Croatiaines.trkulja@gmail.com (I.T.); marija.ivk@gmail.com (M.I.); nikolinajurjevic@yahoo.com (N.J.);; 2 Department of General and Sports Traumatology and Orthopedics, Merkur University Hospital, 10000 Zagreb, Croatia; 3 School of Medicine, University of Zagreb, 10000 Zagreb, Croatia

**Keywords:** rotator cuff calcific tendinitis, Gartner, acromion index, critical shoulder angle, lateral acromial angle, acromiohumeral interval

## Abstract

**Background/Objectives:** Rotator cuff calcific tendinitis (RCCT) is a common cause of shoulder pain. The role of acromial morphology in RCCT pathogenesis remains unclear. This study aimed to evaluate association between acromial morphological parameters and calcific deposit characteristics in patients with RCCT. **Methods:** We retrospectively analyzed 1185 patients who underwent shoulder radiography between January 2015 and January 2025 at Merkur University Hospital, Zagreb, Croatia. After excluding 281 radiographs of insufficient quality, 904 patients (503 females, 401 males; mean age 57.5 ± 13.6 years) were included. Calcific deposits were classified according to Bosworth and Gartner–Heyer systems. Acromial morphology was assessed using the acromion index (AI), critical shoulder angle (CSA), lateral acromial angle (LAA), and acromiohumeral interval (AHI). Non-parametric statistical tests were used with statistical significance set at *p* < 0.05. **Results:** The mean deposit diameter was 13.29 mm. According to Gartner–Heyer classification, 295 patients had type 1, 339 type 2, and 270 type 3 deposits. Significant correlations were found between deposit size and CSA (ρ = −0.08, *p* < 0.05), and AHI (ρ = 0.12, *p* < 0.001), while AI correlated with Gartner–Heyer type (ρ = 0.09, *p* < 0.01). No significant correlations were found for LAA. Kruskal–Wallis testing showed significant differences across deposit groups for AI, AHI, and LAA. **Conclusions:** Acromial morphology is significantly associated with calcific deposit characteristics in RCCT, supporting a potential biomechanical role in disease manifestation. These findings may refine diagnostic assessment and warrant further prospective validation.

## 1. Introduction

Rotator cuff calcific tendinitis (RCCT) is caused by the disposal of hydroxyapatite crystals (Ca_10_(PO_4_)_6_(OH)_2_) in the tendons of the rotator cuff. It is characterized by recurrent episodes of pain and a reduced range of motion of the affected shoulder. The supraspinatus muscle tendon is the most commonly affected (76–80% of patients) [1,2]. The calcification is typically located focally in the tendon, 1–2 cm from its insertion, at the site of maximal mechanical load. According to Souge et al. [3], in 96% of cases, it is a place near the connection of the supraspinatus and infraspinatus tendons. In 15% of patients, the lower side of the infraspinatus tendon is affected, while calcification in the pre-insertional tendon segment of the subscapularis occurs in approximately 5% of cases [4].

RCCT is a common condition with a prevalence ranging from 2.7% to 22% among asymptomatic individuals, and it accounts for 10% to 42% of all shoulder pain cases [5,6,7]. The condition typically affects individuals between the ages of 30 and 60, with women being affected twice as often as men, particularly between the ages of 30 and 50 [8]. It is slightly more common in the dominant hand, but the nondominant hand can also be affected. Interestingly, 10–15% of patients experience bilateral involvement [9]. Notably, RCCT is not linked to strenuous work or sports activity, even in those exposed to higher loads, such as lifting weights overhead. In fact, it appears to affect sedentary individuals more frequently [4,7,10].

The pathogenesis of RCCT remains incompletely understood. Two main speculative theories are usually discussed. The oldest of these was proposed by Codman [11], who believed that degenerative changes in tendon fibers ultimately lead to calcification. Many other researchers support this theory. The representative of the second, so-called multiphasic theory [12] or reactive theory, is Uhthoff, who does not consider RCCT to be a degenerative process [13,14]. According to him, RCCT is a reactive calcification within an otherwise healthy tendon. The process begins with fibrocartilaginous metaplasia of tenocytes, resulting from metabolic and mechanical changes within the tendon. It is followed by a formative phase, a resting phase, a resorption phase, and finally a post-calcification stage during which the tendon is renewed. It is believed that this process differs from those observed in calcifications elsewhere in the body [15]. In degenerative tendinopathies, which most often occur in individuals over 60 years of age, calcifications made up of a mixture of calcium salts are typically spread throughout the tendon [16]. A more recent theory, which supports Uhthoff’s reactive theory, was proposed by Zhang and Wang [17] in 2010. They believe that errors in the differentiation of tendon stem cells (TSCs) can lead to chondral metaplasia and ectopic ossification. Typically, TSCs aid in regenerating tenocytes, particularly after mechanical damage to the tendon.

Despite numerous studies conducted from various perspectives, none of the existing theories have yet proven to be entirely acceptable or provided a definitive and clear explanation for the occurrence of calcifications in the tendons of the rotator cuff. In fact, according to Sansone et al. [9], new evidence supporting theories about the biological and genetic basis of RCCT development further complicate the understanding of the disease’s progression. However, these authors hope that recent positive findings will lead to improved prevention and treatment. Etiologically, several factors are linked to or may contribute to the development of calcifying tendinitis. They are typically categorized as internal or external, but often involve a combination of both. Therefore, RCCT is associated with age, gender, morphological features of the shoulder joint, and various endocrine and metabolic disorders.

Several studies [18,19,20,21] have attempted to identify differences in acromion morphology between individuals with rotator cuff pathologies and those without. Nyffeler et al. [22] showed that rotator cuff tendon rupture is linked to the acromion index (AI), especially the lateral extension of the acromion. However, calcifying tendinitis was generally not studied.

This study aimed to investigate the correlation between the morphological features of the acromion and the characteristics of calcifications in individuals with RCCT.

## 2. Materials and Methods

This retrospective study (January 2015–January 2025) included a total of 1185 patients with the diagnosis of RCCT who were referred to radiographic imaging of their painful shoulder at the Radiology Department at Merkur University Hospital in Zagreb, Croatia. Imaging was conducted on Shimadzu Radspeed Saphire CH200 (Shimadzu Corporation, Kyoto, Japan).

**Figure 1:**
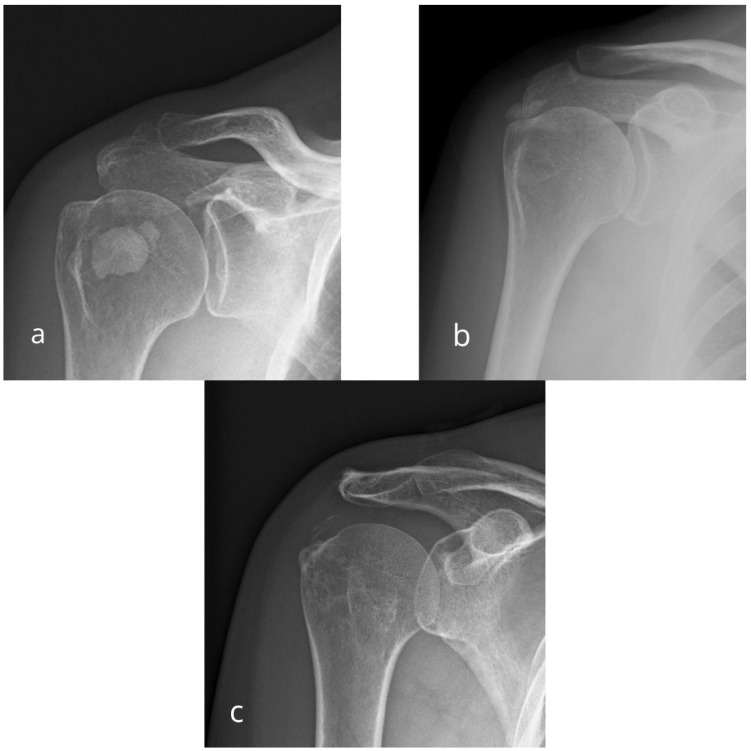
Anteroposterior radiographic views of right shoulder in different patients showing different types of calcific deposits according to the Gärtner–Heyer classification: (**a**) Type I (homogeneous, well-defined)—dense, sharply delineated deposit, (**b**) Type II (partly homogeneous)—mixed-density deposit with partial resorption, (**c**) Type III (inhomogeneous)—poorly defined, low-density deposit indicating the resorptive phase.

Descriptions of calcific deposits were provided using the classification proposed by Gärtner and Heyer [23]. Representative examples of Gartner–Heyer calcific deposit types are shown in Figure 1. The deposits’ longer diameter was measured, and they were accordingly classified using the Bosworth classification [24]. When there was more than one deposit, the diameters were summed.

The morphological features of the acromion were described using the acromion index (AI), critical shoulder angle (CSA), lateral acromial angle (LAA), and acromiohumeral interval (AHI). An experienced MSK radiologist conducted secondary analysis of the above-mentioned morphological features (more than 10 years of practice).

**Figure 2:**
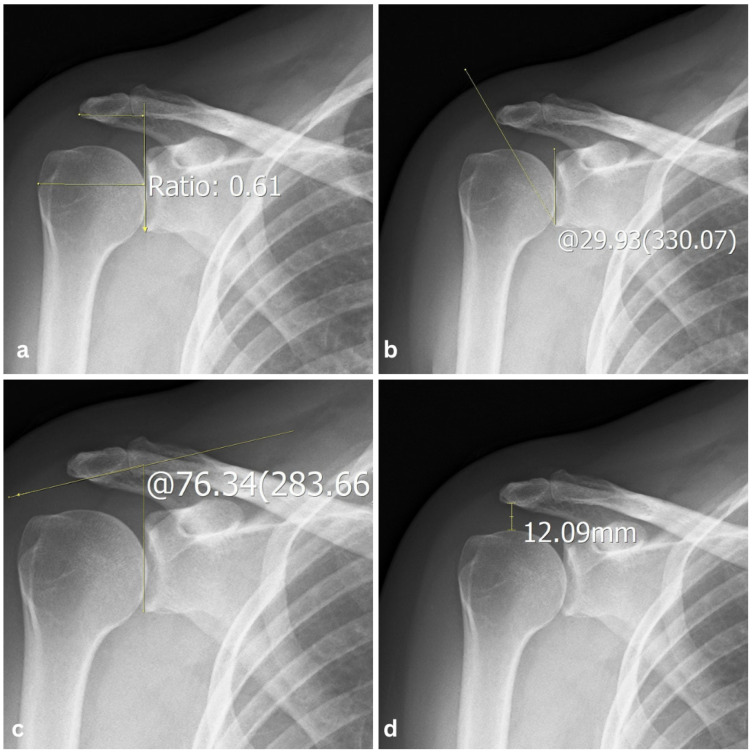
Anteroposterior radiographic view of the right shoulder with measurements examples of: (**a**) acromion index measuring 0.61, (**b**) critical shoulder angle measuring 29.93°, (**c**) lateral acromial angle measuring 76.34°, and (**d**) acromiohumeral interval measuring 12.09 mm.

The shoulder was imaged in two projections, a true anteroposterior projection and in external rotation. The measurements were obtained from the true anteroposterior images. The AI was measured as the ratio between the distance from the glenoid cavity surface to the lateral edge of the acromion and the distance from the glenoid cavity surface to the lateral edge of the humeral head (Figure 2a). The CSA was measured as the angle between the glenoid cavity surface and the most inferolateral border of the acromion (Figure 2b). The LAA was measured as the angle between the glenoid cavity surface and the acromion undersurface (Figure 2c). The AHI was calculated as the distance between the inferior acromion surface and the superior aspect of the humeral head (Figure 2d).

Of 1185 patients, 281 were excluded from the study because their radiographs were of insufficient quality for analysis. Radiographs were excluded if the projection deviated from a true anteroposterior orientation, if the glenoid margins or acromial borders were obscured, or if motion or exposure artifacts precluded accurate angle or distance measurement. Only true anteroposterior shoulder radiographs were included, defined by near-complete overlap of the anterior and posterior glenoid rims (≥50%), corresponding to less than approximately 10–15° of scapular rotation relative to the beam. This tolerance is consistent with previously reported thresholds ensuring measurement reliability of the critical shoulder angle and acromion index [22,25,26]. All images were visually inspected for adequacy by the same musculoskeletal radiologist prior to analysis, and any ambiguous cases were jointly reviewed with a second experienced radiologist to ensure consistency.

To assess intra-observer reliability, 60 randomly selected radiographs were re-evaluated by the same radiologist after a 2-week washout period, with the images re-randomized and the reader blinded to prior values. Each radiographic parameter was measured twice under identical conditions. Intra-observer reliability was quantified using Cronbach’s alpha in Statistica for Windows, Version 14.3 (TIBCO Software Inc., Palo Alto, CA, USA). Cronbach’s alpha values above 0.80 were interpreted as indicating good reliability, and values above 0.90 as excellent reliability.

Statistical analysis was performed using Jamovi Computer Software, Version 2.7.5 (The jamovi project, Sydney, Australia, retrieved from https://www.jamovi.org, accessed on 1 August 2025). We included descriptive statistics, such as measures of central tendency (e.g., the arithmetic mean and median), as well as measures of dispersion (e.g., standard deviation, minimum, and maximum values). The normality of the data distribution was tested using the Shapiro–Wilk test, which revealed a significant deviation from normality for all analyzed variables. Therefore, we applied suitable non-parametric tests for further analysis, such as the Kruskal–Wallis test for comparisons among multiple independent groups. A *p* < 0.05 was considered statistically significant in all tests. All statistical tests were two-tailed. Effect sizes were interpreted according to established conventions (small ≈ 0.01, medium ≈ 0.06, large ≈ 0.14 for ε^2^; weak < 0.30 for correlation coefficients). Spearman’s rank correlation coefficients (ρ) with corresponding 95% confidence intervals (CIs) were calculated to evaluate associations between acromial parameters and calcific deposit characteristics. A *p*-value < 0.05 was considered statistically significant.

Because this was a retrospective study that included all consecutive patients who met the inclusion criteria within the defined 10-year period, no formal sample size calculation was performed. However, the large final cohort (*n* = 904) provides adequate statistical power and representativeness.

## 3. Results

A total of 904 patients were included in the study, with 503 females and 401 males. The average age was 57.49 ± 13.58, ranging from 20 to 91 years (females: 58.07 ± 13.49; males: 56.76 ± 13.68). The right shoulder was affected in 471 patients (52.10%). Intra-observer reliability for radiographic and classification measurements was good to excellent. Cronbach’s α values were 0.97 for AHI, 0.96 for AI, 0.97 for CSA, and 0.86 for LAA. The Gartner–Heyer and Bosworth classifications demonstrated excellent agreement (α = 0.96 and 1.00, respectively).

**Figure 3:**
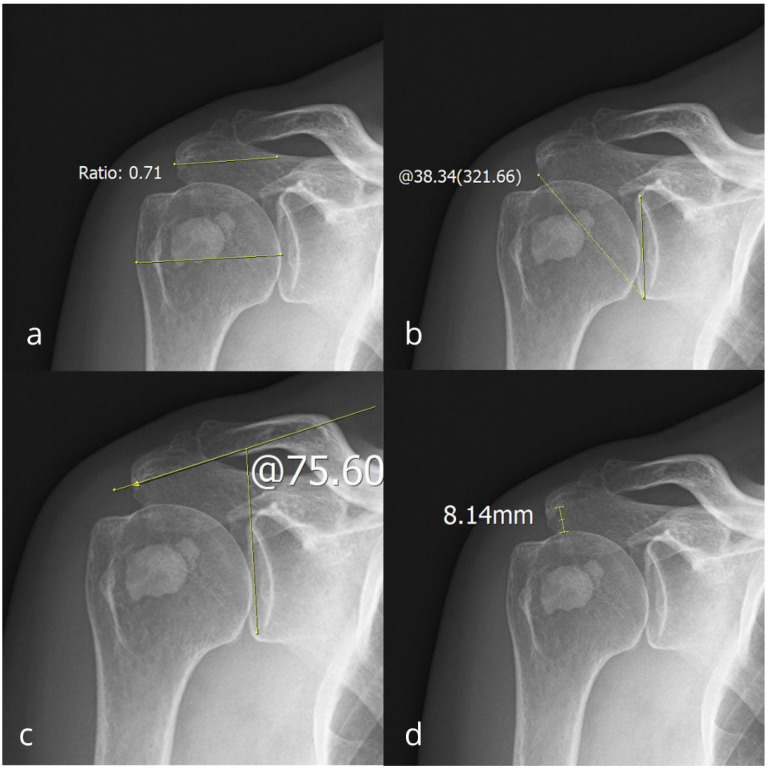
Anteroposterior radiographic view of the right shoulder of a patient with rotator cuff calcific tendinitis with measurements of: (**a**) acromion index measuring 0.71, (**b**) critical shoulder angle measuring 38.34°, (**c**) lateral acromial angle measuring 75.60°, and (**d**) acromiohumeral interval measuring 8.14 mm.

The mean deposit diameter was 13.29 mm (range, 1.00 to 58.00), with a median of 11.00 mm. According to Bosworth’s classification of deposits, 144 patients had small deposits, 450 had medium deposits, and 310 had large deposits. The Gartner-Heyer classification revealed 295 patients with dense, well-circumscribed or type 1 deposits, 339 patients with type 2 or cloudy forms characterized by ill-defined borders, and 270 patients in a resorptive state, exhibiting indistinct borders, relative translucency, or type 3 deposits. Table 1 shows the descriptive statistical values of the examined parameters. CSA mean was 35.11 ± 4.71°, LAA mean was 92.29 ± 9.31°, AI mean was 0.72 ± 0.08, identical to the median, and AHI mean was 8.56 ± 2.33 mm. An example of acromial measurements from a patient with RCCT is shown in Figure 3.

Spearman’s ρ correlation coefficients are shown in Table 2. Almost all acromion descriptors are significantly correlated with calcific deposit size (LAA excluded), while Gartner-Heyes typing correlates significantly only with AI.

Kruskal–Wallis ANOVA test results for comparing three different groups based on the Gartner-Heyer classification, along with post hoc Dwass-Steel-Critchlow-Fligner pairwise comparisons, are presented in Table 3 and Table 4, respectively. Similarly, the same analysis was conducted to test for differences between various sizes of calcific deposits divided into three groups, as Bosworth suggested. Those results are presented in Table 5 and Table 6.

Several variables reach the level of statistical significance due to the presence of statistically significant differences. Significant differences are observed for LAA between Gartner types 1 and 2, and for AI between Gartner types 1 and 3, though with small effect size.

Bosworth categorized calcific deposits into three sizes: small, medium, and large. Analyzing these categories revealed significant differences in AI between medium and large deposits, as well as in AHI between small and large deposits, and between medium and large deposits, though with a small effect size.

## 4. Discussion

RCCT is a common cause of shoulder pain, characterized by calcium deposition within the rotator cuff tendons, most frequently the supraspinatus. It typically affects adults between the ages of 30 and 60 years, often causing pain and restricted motion. In our cohort, the female predominance and mean patient age in the fifth decade were consistent with previous epidemiologic reports [27,28,29,30,31,32,33,34,35].

Several studies have also noted that women tend to experience more severe symptoms, particularly greater pain intensity [7]. De Witte et al. [34] reported that symptoms most often occur when the diameter of the calcification exceeds 10 mm, suggesting that deposit size may influence symptom severity. However, in our study, the median deposit diameter was 11 mm in both sexes, indicating no sex-related difference.

The right shoulder was affected in slightly more than half of patients (52.3%), consistent with earlier studies reporting right-sided involvement in 54–66% of cases [34,36,37,38]. Nevertheless, RCCT is not associated with increased or repetitive use of the affected arm in occupational or athletic activities [39]. In fact, several studies have found it more frequently in sedentary individuals [7,10].

This study investigated the relationship between acromial morphology and the radiographic characteristics of calcific deposits in a large cohort of patients with RCCT. We found that specific acromial parameters, particularly the AI and the CSA, were significantly but weakly correlated with deposit size and type, suggesting that subtle variations in acromial anatomy may influence local tendon loading conditions and subsequent calcium deposition.

The morphology of the acromion is linked to many rotator cuff diseases, and various biomechanical studies suggest a complex interaction between deltoid muscle forces and the rotator cuff [40]. Still, the relationship between acromion shape and RCCT has not been extensively studied. Our results showed significant correlations between the calcific deposit size and its classification by Gartner-Heyer with CSA, AI, and AHI.

From a biomechanical standpoint, the acromial parameters analyzed in this study directly influence the orientation and magnitude of the deltoid muscle vector as well as the dimensions of the subacromial space. An increased CSA and AI reflect greater lateral extension of the acromion, which augments the vertical component of the deltoid force and increases compressive load on the supraspinatus tendon [22,41]. Conversely, a smaller LAA corresponds to a more downward-sloping acromion, which further reduces subacromial clearance and promotes impingement [42,43]. These mechanical alterations may create localized hypoxia and repetitive micro-compression at the tendon–bone interface, predisposing to the fibrocartilaginous metaplasia and calcium deposition typical of RCCT. Thus, the observed morphological associations could support the hypothesis that subtle variations in acromial anatomy contribute to pathological tendon loading within the subacromial impingement continuum. Taken together, these findings provide biomechanical support for the modest but significant correlations observed in our study.

The average and median values of AI in our patients were both 0.72. It is higher than the values reported in most other studies. For example, Prietzel and colleagues [44] recently established normative AI values through a comprehensive MRI study, which were notably lower (0.52 on the right and 0.50 on the left). Balke et al. [45] examined differences in acromion morphology between healthy individuals and those with RCCT. They found that AI was higher in patients with RCCT, matching the mean values in our study, and also higher in those with supraspinatus tendon rupture. Nyffeler et al. [22] reported higher AI in patients with full-thickness rotator cuff tears, whereas values in patients with osteoarthritis and controls were significantly lower. Nyffeler et al. [22] explained that AI measures the lateral extension of the acromion above the humeral head, suggesting that greater lateral extension might predispose the supraspinatus tendon to degeneration. Theoretically, this extension could influence the development of calcific deposits. In addition to AI, Balke et al. [45] also evaluated LAA. Unlike our findings, they discovered that LAA in patients with RCCT was significantly narrower than in controls. This was confirmed in the investigation by Birsel et al. [40] ten years later. However, in our study, LAA was larger than described by Balke or Birsel, and it was the only parameter that showed no correlation with deposit size or the Gartner-Heyer classification.

Kirscher et al. [46] found no relationship between AI and the severity of symptoms; high AI levels did not influence pain severity or functional impairment in the shoulder joint for individuals with calcification in the supraspinatus tendon. They also did not find a statistically significant correlation with the size of the calcification or any other classification of calcifications.

The CSA is one of the newest attributes used to describe acromion morphology. It combines measurements of glenoid inclination and the lateral extension of the acromion on anteroposterior radiographs. Heuberer et al. [47] concluded in their study that CSA, along with patients’ age, effectively predicts various shoulder pathologies, including RCCT. Similarly, Hsu et al. [48] found that CSA can predict supraspinatus tendinopathy. In our study, CSA showed correlations comparable to AI with deposit size and the Gartner-Heyer classification. Its values were slightly higher than those reported by Heuberer et al., but lower than those reported by Hsu et al. Hsu indicated that a CSA of 38.11° is the acceptable threshold for predicting supraspinatus tendinopathy. Overall, CSA has been mainly studied in relation to rotator cuff tears at different stages [49,50], and the CSA values observed were higher than those in our patients.

Despite the correlation with deposit size and the Gartner-Heyer classification, no significant differences were observed between the different stages of the Gartner-Heyer or Bosworth classifications.

Ricci et al. [51] described the AHI as a specific anatomical region between the deep surface of the deltoid muscle and the superficial part of the rotator cuff, where fat tissue, lax connective tissue, SASD synovial bursa, and subdeltoid fascia are located. It is generally defined as the vertical distance between the lower surface of the acromion and the superior aspect of the humeral head on AP radiographs. As Brolin et al. [52] stated, the interval in healthy shoulders typically ranges from approximately 7 to 11 mm. The mean values of our patients fell within this range but were closer to the lower end. Several studies have shown that a narrowed AHI is a strong indicator of rotator cuff tears [52,53], but it also appears to be associated with RCCT.

For AHI, we observed the strongest correlation with deposit size, whether measured by diameter or the Bosworth classification. It was also noted that, considering the Bosworth classification of deposit size, AHI results show the most significant differences between grades, specifically between small and large deposits, as well as between medium and large deposits.

In conclusion, although the correlations between acromial parameters and calcific deposit characteristics reached statistical significance, their magnitude was weak (ρ = 0.08–0.12). This suggests that acromial morphology exerts only a modest influence on deposit formation and distribution. The weak effect sizes are consistent with the multifactorial nature of rotator cuff calcific tendinopathy, which likely results from the interplay of mechanical, metabolic, and cellular processes rather than a single anatomic determinant. Therefore, while acromial configuration may predispose to altered tendon loading, it should be interpreted as one of several interacting factors rather than a primary cause.

This study has several limitations that should be acknowledged. First, its retrospective and single-center design may introduce selection bias and limit the generalizability of the findings. Because this study was conducted at a single institution in Zagreb, Croatia, and the study population was relatively homogeneous, potential racial or ethnic differences could not be assessed. Therefore, the generalizability of our findings to other populations may be limited; future multicenter studies, including ethnically diverse cohorts, are warranted. Second, all measurements were obtained from plain radiographs, which, although widely available and standardized, provide only two-dimensional projections and are susceptible to positioning errors despite the exclusion of inadequate images. Third, the cross-sectional design enables us to demonstrate associations between acromial morphology and calcific deposit characteristics, but it does not permit causal inference. Furthermore, we did not include clinical parameters such as pain severity, functional outcomes, or symptom duration, which may have provided additional insights into the clinical significance of these anatomical variations. Clinical data such as comorbidities (e.g., diabetes mellitus, thyroid disease) and lifestyle factors (e.g., smoking status) were unavailable in the radiographic database and could therefore not be analyzed. As these variables may influence tendon homeostasis and calcification, their inclusion in future prospective studies would provide a more comprehensive understanding of RCCT pathogenesis. Despite these limitations, the large sample size and consistent measurement methodology strengthen the validity of our results, highlighting the potential role of acromial anatomy in the pathogenesis of RCCT.

## 5. Conclusions

This study demonstrates that morphological features of the acromion are significantly associated with the characteristics of calcific deposits in patients with RCCT. These findings suggest that radiographic evaluation of acromial morphology may provide additional insight into the pathophysiology of RCCT, helping to refine diagnostic and therapeutic strategies. Further prospective studies integrating advanced imaging and longitudinal follow-up are warranted to confirm these associations and clarify their clinical implications.

## Figures and Tables

**Table 1:** Descriptive statistical values of the examined parameters.

		95% Confidence Interval						Shapiro–Wilk	
	Mean	Lower	Upper	Median	SD	Min	Max	W	*p*
CSA	35.11	34.80	35.41	34.80	4.71	23.69	48.65	0.91	<0.001
LAA	92.29	91.68	92.90	93.24	9.31	66.25	118.52	0.99	<0.001
AI	0.72	0.71	0.72	0.72	0.08	0.48	1.20	0.99	<0.001
AHI	8.56	8.41	8.71	8.00	2.33	2.00	18.00	0.98	<0.001

**Table 2:** Correlation matrix—Spearman’s ρ coefficients.

	Deposit Size	CSA	LAA	AI	AHI	Bosworth	Gartner
Deposit size	—						
CSA	−0.08 *	—					
CI lower	−0.14						
upper	−0.02						
LAA	0.01	−0.32 ***	—				
CI lower	−0.06	−0.38					
upper	0.08	−0.28					
AI	−0.05	0.55 ***	0.12 ***	—			
CI lower	−0.12	0.50	0.06				
upper	0.02	0.59	0.18				
AHI	0.12 ***	−0.50 ***	0.20 ***	0.11 ***	—		
CI lower	0.06	−0.55	0.14	0.05			
upper	0.18	−0.45	0.26	0.17			
Bosworth	0.90 ***	−0.08 *	0.01	−0.08 *	0.12 ***	—	
CI lower	0.89	−0.14	−0.06	−0.14	0.08		
upper	0.91	−0.02	0.08	−0.02	0.18		
Gartner	0.10 *	0.00	0.05	0.09 **	−0.00	0.09 **	—
CI lower	0.04	−0.07	−0.02	0.03	−0.07	0.03	
upper	0.16	0.07	0.12	0.15	0.07	0.15	

* *p* < 0.05, ** *p* < 0.01, *** *p* < 0.001.

**Table 3:** Kruskal–Wallis test for groups in accordance with Gartner-Heyer classification.

	χ^2^	df	*p*	ε^2^
CSA	0.86	2	0.652	9.49 × 10^−4^
LAA	8.15	2	0.017	0.00906
AI	7.68	2	0.021	0.00851
AHI	3.90	2	0.142	0.00433

ε^2^ = effect size for the Kruskal–Wallis test; small ≈ 0.01, medium ≈ 0.06, large ≈ 0.14.

**Table 4:** Post hoc Dwass-Steel-Critchlow-Fligner pairwise comparisons.

Gartner		CSA		LAA		AI		AHI	
		W	*p*	W	*p*	W	*p*	W	*p*
1	2	−1.10	0.752	4.02	0.012	0.88	0.807	2.36	0.218
1	3	0.12	0.996	1.94	0.354	3.71	0.024	−0.20	0.990
2	3	1.20	0.672	−1.96	0.347	3.04	0.081	−2.40	0.206

**Table 5:** Kruskal–Wallis test for groups categorized according to the Bosworth classification.

	χ^2^	df	*p*	ε^2^
CSA	5.62	2	0.060	0.00624
LAA	0.44	2	0.801	4.93 × 10^−4^
AI	8.77	2	0.012	0.00972
AHI	12.54	2	0.002	0.01391

ε^2^ = effect size for the Kruskal–Wallis test; small ≈ 0.01, medium ≈ 0.06, large ≈ 0.14.

**Table 6:** Post hoc Dwass-Steel-Critchlow-Fligner pairwise comparisons.

Bosworth		CSA		LAA		AI		AHI	
		W	*p*	W	*p*	W	*p*	W	*p*
1	2	−1.24	0.655	−0.43	0.950	0.60	0.905	2.36	0.217
1	3	−3.05	0.079	0.35	0.967	−2.62	0.153	4.58	0.003
2	3	−2.57	0.164	0.91	0.795	−4.06	0.011	3.59	0.030

## Data Availability

Data is contained within the article, and further inquiries can be directed to the corresponding author.
